# Progress and pitfalls of gene editing technology in CAR-T cell therapy: a state-of-the-art review

**DOI:** 10.3389/fonc.2024.1388475

**Published:** 2024-06-07

**Authors:** Vahid Moradi, Elnaz Khodabandehloo, Mehdi Alidadi, Azadeh Omidkhoda, Naser Ahmadbeigi

**Affiliations:** ^1^ Hematology and Blood Transfusion Science Department, School of Allied Medical Sciences, Tehran University of Medical Sciences, Tehran, Iran; ^2^ Department of Immunology, School of Medicine, Hamadan University of Medical Sciences, Hamadan, Iran; ^3^ Department of Anatomy, School of Medicine, Tehran University of Medical Sciences, Tehran, Iran; ^4^ Gene Therapy Research Center, Digestive Disease Research Institute, Tehran University of Medical Sciences, Tehran, Iran

**Keywords:** chimeric antigen receptor (CAR), gene editing, CRISPR, CAR-T cell, immunotherapy, cancer

## Abstract

CAR-T cell therapy has shown remarkable promise in treating B-cell malignancies, which has sparked optimism about its potential to treat other types of cancer as well. Nevertheless, the Expectations of CAR-T cell therapy in solid tumors and non-B cell hematologic malignancies have not been met. Furthermore, safety concerns regarding the use of viral vectors and the current personalized production process are other bottlenecks that limit its widespread use. In recent years the use of gene editing technology in CAR-T cell therapy has opened a new way to unleash the latent potentials of CAR-T cell therapy and lessen its associated challenges. Moreover, gene editing tools have paved the way to manufacturing CAR-T cells in a fully non-viral approach as well as providing a universal, off-the-shelf product. Despite all the advantages of gene editing strategies, the off-target activity of classical gene editing tools (ZFNs, TALENs, and CRISPR/Cas9) remains a major concern. Accordingly, several efforts have been made in recent years to reduce their off-target activity and genotoxicity, leading to the introduction of advanced gene editing tools with an improved safety profile. In this review, we begin by examining advanced gene editing tools, providing an overview of how these technologies are currently being applied in clinical trials of CAR-T cell therapies. Following this, we explore various gene editing strategies aimed at enhancing the safety and efficacy of CAR-T cell therapy.

## 1 Introduction

As the first gene-manipulated cell-based product approved for immune-oncology treatment, chimeric antigen receptor T cells (CAR-T cells) have led to major successes in the treatment of CD19^+^ B-cell malignancies and multiple myeloma. Since Kymriah was approved by the FDA in 2017, seven other products have been approved by different regulatory authorities to enter the market (five by the FDA, one by the State Food and Drug Administration of China, and one by the Spanish Agency of Medicines and Medical Devices) (1). Nevertheless, several hurdles and complexities limit its use as a first-line treatment (2). Furthermore, due to challenges such as the selection of appropriate target antigen, CAR-T cell trafficking into tumor sites, and immunosuppressive tumor microenvironment, CAR-T cell therapy has so far not been effective in the treatment of solid tumors and non-B cell hematologic malignancies (3, 4). In recent years, many efforts have been made to unlock the current bottlenecks of CAR-T cell therapy and move CAR-T cells to the first line of cancer treatment. These efforts include the selection of suitable target antigen for CAR-T cells (5), optimization of T cell activation and expansion methods (6), optimization of CAR structure (7), and comparing different types of vectors and delivery systems to choose the safest and most efficacious ones (8).

The emergence of gene editing technologies specifically clustered regularly interspaced short palindromic repeats and CRISPR-associated protein 9 (CRISPR/Cas9) system have opened a new way in the development and optimization of CAR-T cell therapy. Most of the exorbitant costs of CAR-T cell therapy are related to the quality control tests as well as the use of retroviral/lentiviral vectors. Compared to viral vectors that have a time-consuming production process and various complex and expensive molecular and biochemical quality control tests, the CRISPR/Cas9 system is easy to design and has relatively simpler quality control tests (9). Gene editing tools make it possible to insert transgenes with small sizes including CAR into safe genomic harbors within the host genome. Thus, gene editing tools can provide a virus-free knock-in system that enables more controlled gene insertion while avoiding the high costs and side effects of viral vectors (10). Cas9 and other programmable nucleases can be transferred into the cells in the form of DNA, RNA, or Ribonucleoprotein (RNP) by various delivery systems such as electroporation (11). Additionally, the genome-wide applicability of gene editing tools to target any desired gene within the human genome has paved the way to achieving an “off-the-shelf” CAR-T product and further increasing the safety and efficacy of CAR-T cell therapy (12).

Although gene editing has revolutionized CAR-T cell therapy, the use of classical gene editing tools [Zinc finger nucleases (ZFNs), Transcription activator-like effector nucleases (TALENs), and CRISPR/Cas9] carries a significant risk of off-target activity and genotoxicity (13). Accordingly, numerous initiatives have been undertaken to lessen the risk of genotoxicity which has resulted in the development of advanced gene editing tools with higher precision and improved safety profile. In the initial section, we review the latest advancements in gene editing tools designed for high-precision genetic modifications, highlighting their roles in CAR-T cell therapy clinical trials. The subsequent section delves into various gene editing strategies aimed at enhancing both the safety and effectiveness of CAR-T cell therapies.

## 2 Gene editing tools for CAR-T cell therapy

### 2.1 Classical tools

Zinc finger nucleases (ZFN), Transcription activator-like effector nuclease (TALEN), and CRISPR/Cas9 are the three types of classical gene editing tools. Unlike ZFNs and TALENs which are man-made artificial tools, CRISPR/Cas9 is a naturally occurring system, based on an optimized version of the Streptococcus pyogenes antiviral defense system (14). ZFN comprised arrays of engineered zinc finger domains and a bacterial Fok1 nuclease. Zinc finger domains specifically bind to the target DNA region and enable Fok1 to induce a double-strand break (DSB) in the target site (15).

TALEN consists of site-specific DNA binding TALE domain and a non-specific DNA cleavage Fok1 nuclease (16). TALE is a naturally occurring protein of Xanthomonas bacteria that comprises tandem arrays of highly conserved 33–34 amino acid repeats. The 12^th^ and 13^th^ amino acids are highly variable and called repeat-variable di-residues that cause binding to one of the four types of nucleotides (17). TALENs elicit double-strand breaks (DSBs) within the designated loci, similar to ZFNs. Unlike ZFNs which require linkage between zinc finger domains, in TALENs, each TALE repeat has a distinct DNA binding specificity and is designed independently. Therefore, the design and reprogramming of TALENs is more straightforward than ZFNs (14, 18).

The discovery of the CRISPR/Cas9 system was a game changer in the field of genome editing. In contrast to ZFNs and TALENs which bind to target sequences through protein-DNA interactions, in the CRISPR/Cas9 system recognition of target sites is based on RNA-DNA interactions (14). Compared to the design of proteins, which require complex and costly engineering processes, RNA is an easy-to-design molecule. The simple RNA design has facilitated the generation of genome-wide CRISPR/Cas9 libraries to target any desired sequence within the human genome (19–22). CRISPR/Cas9 consists of a non-specific Cas9 nuclease and a single guide RNA (sgRNA) designed to recognize a specific 20-bp sequence within the targeted gene. The sgRNA comprises a 17–20 nucleotide CRISPR RNA (crRNA) that is complementary to the target site and a hybridized trans-acting CRISPR RNA (tracrRNA) which functions as a binding scaffold for Cas9 enzyme (23). To generate a DSB in the target sequence, Cas9 first binds to an NGG nucleotide sequence called the “protospacer adjacent motif” (PAM) at the 3′ end of the target sequence. Cas9 then binds to the non-protospacer part of sgRNA and cleaves the target site (24). Compared to ZFNs and TALENs, multiplex genome editing using CRISPR/Cas9 is more straightforward (14). In this method, different sgRNAs are designed to simultaneously edit different unrelated genes in the same cell. Different characteristics of classical gene editing tools are compared in **Table 1**.

**Table 1:** Comparison of classical gene editing tools.

	ZFN	TALEN	CRISPR/Cas9	References
Size	1kb†	3 kb†	4.2 kb	(14)
Source	Man-made	Man-made	Naturally occurring	(25)
DNA recognition domain	Protein	Protein	RNA	(26)
Target sites length	9–18 bp	30–40 bp	22 bp + PAM sequence	(14)
Design Process	Costly and time-consuming	Costly and time-consuming	Relatively cheaper and simpler	(14)
tolerance for mismatches	Lower than CRISPR/Cas9	Lower than CRISPR/Cas9	High	(27)
Reprogramming	Extremely complex	Complex	Simple	(14)
Immunogenicity	Low	Low	High	(28)
RNA editing	Not applicable	Not applicable	applicable	(29)
Genome-wide library	Not much progress	Accessible	Accessible	(14, 30)
multiplex genome editing	Difficult	Difficult	More feasible	(30)
Difficulties of Delivery into the cells	Relatively easy due to its small size	Challenging due to its large size	Moderate	(27)

†TALENs and ZFNs are delivered into the cells as a pair, doubling their effective size.

**Figure 1:**
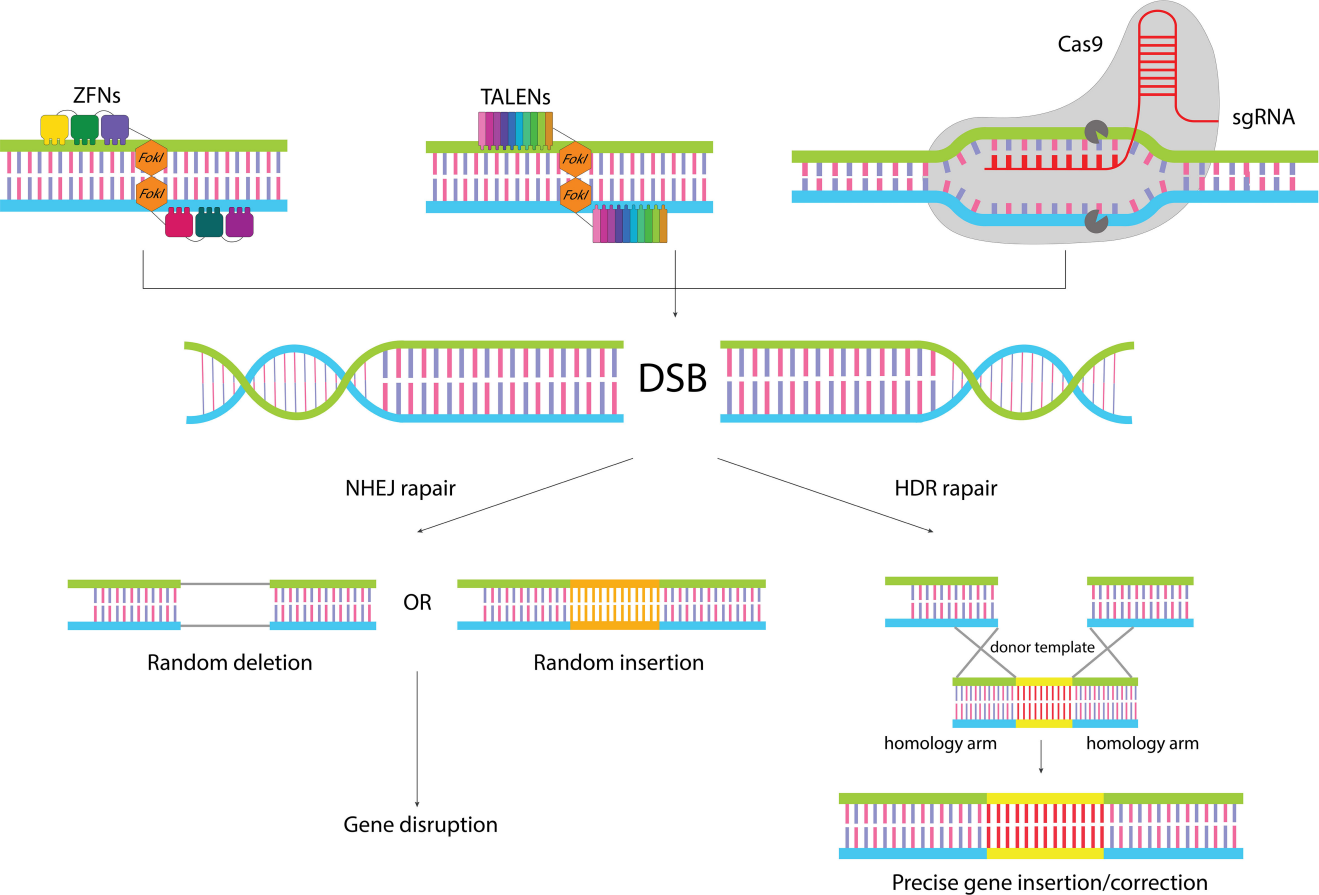
Gene editing by classical gene editing tools. ZFNs and TALENs are modular in shape, and function as dimers where each subunit binds to one of the target site strands, leading to dimerization of Fok1 and cleavage of the target site. In the CRISPR/Cas9 system recognition of the target site is conducted by sgRNA, which makes it easy to reprogram. When the nucleases cleave the target sequence, the generated DSB can be repaired by two different repair pathways: NHEJ pathways which leads to random insertion/deletion and disruption of the gene, or HDR pathway which in the presence of a donor template can lead to precise insertion of gene of interest or correction of target gene.

Genome editing encompasses a wide range of techniques used to alter the DNA of an organism, which can involve modifying multiple genes or large segments of the genome. This contrasts with gene editing, which specifically targets precise changes to individual genes, often to correct mutations or alter gene function. In the realm of gene editing, programmable nucleases play a crucial role. These engineered proteins are designed to create a single double-strand break (DSB) at a specific location within the DNA, directly targeting the gene of interest. The subsequent modification of the genome is not directly enacted by these nucleases but rather relies on the cell’s own DNA repair pathways to address the induced DSB (**Figure 1**). The induced DSB can be repaired by two main DNA repair pathways: nonhomologous end-joining (NHEJ) and homology-directed repair (HDR) (31). NHEJ is the most common repair mechanism, but it is highly imprecise and leads to random insertion/deletion mutations during broken ends ligation, ultimately precipitating gene disruption. In contrast, HDR is a faithful mechanism that uses a homologous template to precisely repair the damaged site (32). The HDR pathway manifests its functionality predominantly during the S- or G2-phase of the cell cycle and utilizes a homologous DNA template to render the repair (33). It can be used to insert the gene of interest into the desired site or correct mutant alleles. For this purpose, a DNA template consisting of homology arms flanking the desired sequence must be transferred to the site of the generated DSB (34). Several efforts have been made to promote the rate of the HDR pathway including genetic or chemical inhibition of NHEJ mediators, employing timed delivery of Cas9, and development of modified Cas9 enzymes (34).

### 2.2 Advanced tools

Although traditional gene editing tools have shown promising results in CAR T cell therapy, they encounter two significant hurdles: first, the potential for genetic aberrations arising from unintended, off-target cuts in the DNA; and second, it is challenging to perform gene editing in resting (non-activated) T cells (35). The discovery of CRISPR/Cas9 was a revolution in the era of gene editing, as the CRISPR system offers a simple and cost-effective approach to target any desired sequence and there are multiple software to predict possible off-target sites. Nevertheless, RNA-DNA interaction-based recognition of target sites in the CRISPR system makes it more prone to off-target activity, compared to ZFNs and TALENs (36, 37). However, some studies have shown that by optimizing the designing algorithms to select the best sgRNA the efficiency of CRISPR/Cas9 increases to a level comparable to that of TALENs and ZFNs or even outperforms them (38, 39). Furthermore, there is also the risk of chromosomal abnormalities in the context of both on-target as well as off-target activity, when the generated DSB interacts with another spontaneous or nuclease-induced DSB within the genome (40).

The efficiency of gene editing is mostly affected by gene expression level. Genes that are actively transcribed have a higher potential for effective editing compared to genes that are not actively transcribed. This is attributed to the heterochromatin structure that facilitates the access of nucleases to the genes (41). Most gene editing tools should be used in activated T cells, which can accelerate T cell differentiation. Several studies have revealed that a high proportion of less differentiated T cells, naive (TN), central memory (TCM), and stem-like memory (TSCM) T cells, are associated with good response to CAR T cell therapy. Their less differentiated phenotype leads to their sustained *in vivo* persistence and proliferation, which is associated with a more favorable anti-tumor activity (42).

**Figure 2:**
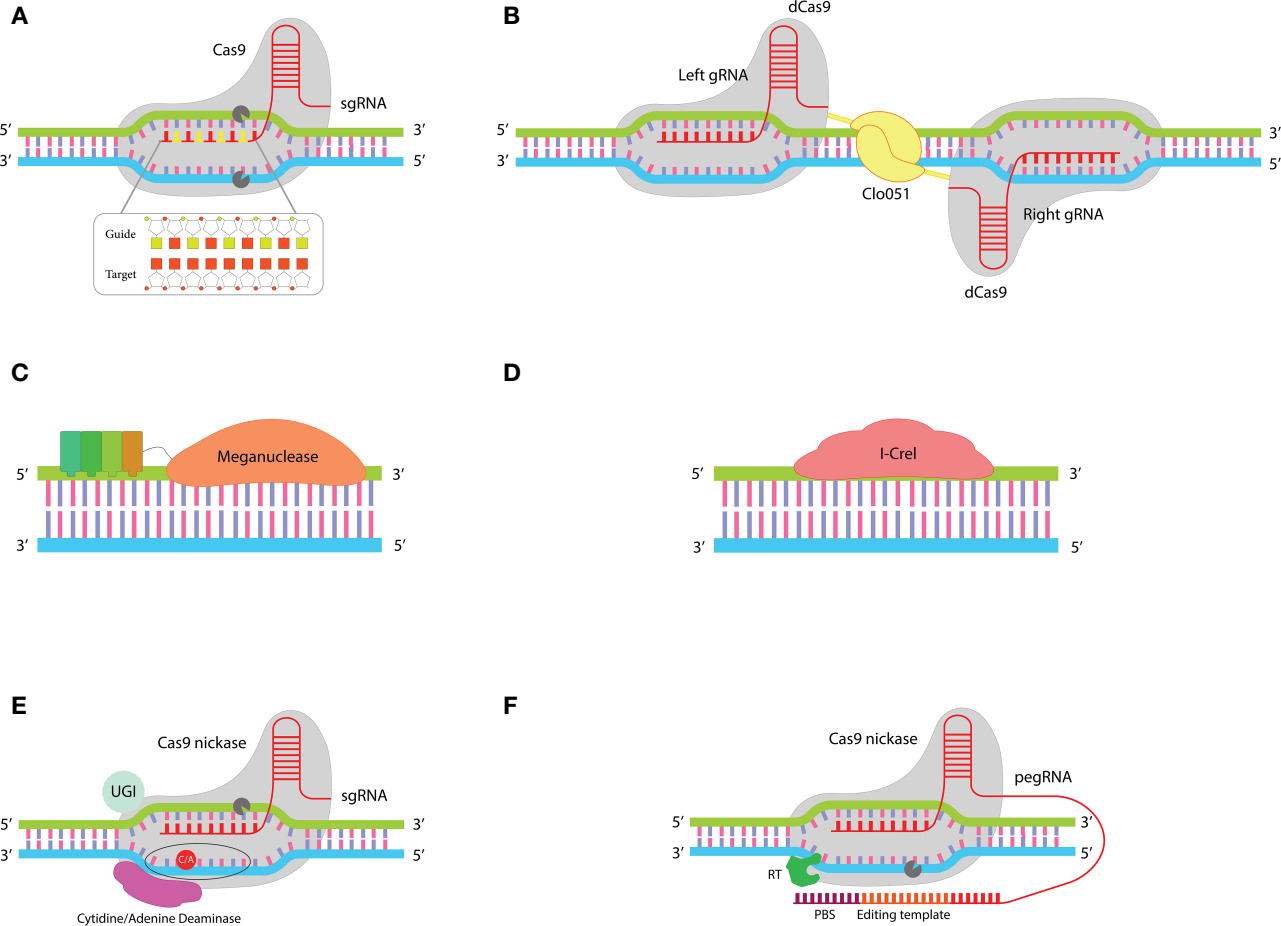
Improved gene editing tools: **(A)** In the chRDNA/Cas9 system the use of a hybrid RNA-DNA guide instead of single guide RNA leads to an increased specificity of Cas9 nuclease. **(B)** The dual guiding strategy in the Cas-CLOVER system increases its fidelity compared to the single-guiding CRISPR system. This system comprises two dCas9 each of which fused to a Clo051 subunit. Activation of Clo051 requires its dimerization which is dependent on guiding each subunit to the target site by a separate sgRNA. **(C)** MegaTAL consists of TALE arrays fused to a meganuclease. This structure sums up the binding affinity of TALE arrays with the site-specific nuclease activity of meganuclease in a single structure. **(D)** ARCUS is a monomeric meganuclease called I-Crel that performs both target site recognition and cleavage. **(E)** Base editors include a sgRNA and a fusion protein (containing a cytidine/adenine base editor linked to nCas9). This structure enables base-to-base changes without introducing DSBs at target sites. **(F)** Prime editors consist of a fusion protein (comprising a reverse transcriptase fused to an nCas9) and a pegRNA (a sgRNA with a PBS and template sequence at the 3’ end). When nCas9 nicks the strand that is complementary to sgRNA, PBS binds to the generated DNA flap and RT reverse transcribes the editing template to DNA that is incorporated into the host DNA at the nicked site.

Based on the demand, various improved tools grounded on the traditional CRISPR-Cas technology and other precise programmable nucleases have been developed in recent years (**Figure 2**) to reduce the risk of off-target effects and chromosomal abnormalities and enable gene editing in resting T cells. These tools will be discussed in the following sections. Clinical trials of genome-edited CAR-T cells and the used tools for their editing are summarized in **Table 2**.

**Table 2:** Clinical trials of genome-edited CAR-T cells.

Tools	CAR-T product	Registration number	Target antigen	Disrupted gens	Study phase
**TALEN**	ALLO-715	NCT04093596	BCMA	*TRAC, CD52*	Phase I
	ALLO-501	NCT03939026, NCT05714345, NCT04416984	CD19	*TRAC, CD52*	Phase I-II
	ALLO-605	NCT05000450	BCMA	*TRAC, CD52*	Phase I
	UCART123	NCT03190278, NCT03203369, NCT04106076	CD123	*TRAC*	Phase I
	UCART22	NCT04150497	CD22	*TRAC, CD52*	Phase I
	UCARTCS1	NCT04142619	CS1	*TRAC, CS1*	Phase I
	UCART19	NCT02735083, NCT02746952, NCT02808442	CD19	*TRAC, CD52*	Phase I
**CRISPR/Cas9**	PACE CART19	NCT05037669	CD19	*TRAC, β2M, CII-TA*	Phase I
	cta30x UCART	NCT05015972	CD20	*TRAC*	Early Phase I
	WU-CART-007	NCT05377827, NCT04984356	CD7	*TRAC, CD7*	Phase I
	CD7 UCAR-T cells	NCT04264078	CD7	*CD7, TRAC*	Early Phase I
		NCT03398967	CD19/CD22/CD20	*TRAC*	phase 1 - phase 2
	anti-mesothelin CAR-T cells	NCT03545815	mesothelin	*TRAC, PD1*	Phase I
	UCART019	NCT03166878	CD19	*TRAC, β2M*	Phase I
	CTX110	NCT04035434	CD19	*TRAC*	Phase I
	CTX112	NCT05643742	CD19	*TRAC, β2M*	Phase I-II
	CTX120	NCT04244656	BCMA	*TRAC, β2M*	Phase I
	CTX130	NCT04502446, NCT04438083	CD70	*TRAC, β2M*	Phase I
	FT819	NCT04629729	CD19	*TRAC*	Phase I
	TT52CAR19	NCT04557436	CD19	*TRAC, CD52*	Phase I
	CT125A	NCT04767308	CD5	*TRAC, CD5*	Phase I
	GC008t	NCT03747965	mesothelin	*PDCD-1*	Phase I
	MPTK-CAR-T	NCT03545815	mesothelin	*PDCD-1, TRAC*	Phase I
	CAR-EGFR T Cells	NCT04976218	EGFR	*TGF-β receptor II*	Phase I
	AJMUC1	NCT05812326	mesothelin	*PDCD-1*	Phase I-II
	XYF19 CAR-T cell	NCT04037566	CD19	*HPK1*	phase I
	UCART123	NCT03203369	CD123	*TRAC*	Phase I
**ARCUS**	PBCAR0191	NCT03666000	CD19	*TRAC*	phase I-II
	PBCAR19B	NCT04649112	CD19	*TRAC, HALE- β2M*	Phase I
	PBCAR20A	NCT04030195	CD20	*TRAC*	phase I-II
	PBCAR269A	NCT04171843	BCMA	*TRAC*	Phase I-II
**ChRDNA/Cas9**	CB-010	NCT04637763	CD19	*TRAC, PDCD-1*	Phase I
**ChRDNA/Cas12a**	CB-011	NCT05722418	BCMA	*TRAC, β2M*, site-specific insertion of *β2M -HLA-E* fusion protein	Phase II
**Cas-CLOVER™**	P-BCMA-ALLO1	NCT04960579	BCMA	*TRBC, β2M*	phase I
	P-MUC1C-ALLO1	NCT05239143	MUC1-C	*TRBC, β2M*	Phase I
**Base editors**	BE-CAR7	ISRCTN15323014	CD7	*TRBC, CD7, CD52*	phase I

TRAC, T cell receptor α constant; TRBC, T cell receptor β constant; PCD-1, Programmed cell death, β2 microglobulin; TGF- β, tumor growth factor β.

#### 2.2.1 CRISPR hybrid RNA-DNA (chRDNA)

One widely explored method to enhance the specificity of Cas9’s activity involves modifying the structure of its sgRNA. While RNA offers precise targeting, it tends to be more expensive to synthesize and less chemically stable compared to DNA. A purely DNA-based guide, however, cannot effectively harness Cas9’s targeting capabilities. First time Rueda et al. showed that Cas9 can be guided to target sites by a hybrid DNA-RNA (chRDNA) guide. They revealed that Cas9 activity is strongly dependent on the presence of ribose at three specific positions of the crRNA that cannot be converted to deoxyribose (43). The distinct advantage of chRDNA compared to traditional sgRNA is the reduction of off-target activities. Compared to RNA-DNA bindings, DNA-DNA bindings have a lower affinity, so placing deoxyribonucleotides between ribonucleotides in the structure of the guide molecule leads to a decrease in the whole affinity between the guide and the target site (**Figure 2A**). This detuned affinity prevents Cas9 activation in cases of mismatches, while if there is a full match between the guide and target site the affinity will be sufficient for Cas9 activation. Therefore, chRDNA dramatically reduces off-targets without disturbing the on-target activity of Cas9 (44–46).

The Cas endonucleases are not limited to Cas9. There are two classes of Cas endonucleases which are different based on their effector nucleases. Class I nucleases have a complex structure with multiple subunits, while class II endonucleases (such as Cas9) are large proteins with a single subunit (47). Cas12a or Cpf1 is a class II type V Cas endonuclease. Cas12a is an RNA-guided endonuclease that creates a DSB at the target site, similar to Cas9 (48). However, there are key differences between Cas9 and Cas12a. The size of the Ca12a is about one-third of the Cas9. Additionally, in contrast to Cas9 which needs both crRNA and tracrRNA, Cas12a only requires a crRNA (49). This smaller size and lack of need for tracRNA simplifies its packaging and facilitates its delivery by various delivery methods such as adeno-associated viral vector, which has a limited gene cargo capacity. Unlike Cas9 which has endonuclease activity, Cas12a is able to cut ribonucleotides in addition to deoxyribonucleotides (48). Cas9 recognizes the G-rich PAM sequence at the 3’ end of the target sites, while Cas12a recognizes the T-rich PAM sequence (TTN or TTTN) at the 5’ upstream of the target sites (49). Furthermore, Cas9 activity creates blunt ends in the cut site, whereas Cas12a activity creates sticky ends (50). This makes the CRISPR/Cas12a system suitable for site-specific insertion of genes of interest or correction of target sequence through the HDR pathway. In addition, Cas12a can cut RNA, a capability that Cas9 lacks (48). Studies have shown that the off-target activity of Cas12a is lower than that of Cas9, but this remains a concern in therapeutics (51–53). It is shown that the use of an RNA-DNA hybrid guide dramatically reduces the off-target activity of Cas12a (54).

CB-010 and CB-011 are two allogeneic CAR T cell products, developed by “Caribou Biosciences” using chRDNA-mediated gene editing of T cells. CB-010 is a CD19 CAR T cell generated using chRDNA-guided Cas9 and harbors three genetic edits: knocking out of *PD-1* gene, disruption of T cell receptor α constant (*TRAC*) gene, and insertion of the CAR transgene into the TRAC locus (55). In CB-011 Cas9 is replaced by Cas12a to make four edits in T cells: site directional insertion of anti-BCMA CAR into the TRAC locus which results in knocking out of the *TRAC* gene, and coupling the disruption of *B2 microglobulin* (*B2M*) with the site-specific insertion of a gene encoding *B2M-HLA-E* fusion protein into the disrupted B2M locus (56). CB-010 and CB-011 are being evaluated in phase I clinical trials for patients with relapsed/refractory B cell non-Hodgkin lymphoma and relapsed/refractory multiple myeloma, respectively (NCT04637763, NCT05722418).

#### 2.2.2 Dead Cas9-X dual-guided nucleases

Cas9 contains two critical endonuclease domains, HNH and Ruv-C, which are responsible for cleaving the strands of DNA that are complementary and non-complementary to the guide RNA (crRNA), respectively. By introducing specific mutations—H840A in the HNH domain and D10A in the Ruv-C domain—Cas9’s ability to cut DNA is inhibited, yet it still maintains the capacity to bind to its target DNA sequence. This altered version of Cas9 is known as partially inactive or dead Cas9 (dCas9) (57). As a result, dCas9 serves as a versatile platform for directing the site-specific delivery of active domains that are attached to it (dCas9-X), leveraging its targeting capability without cleaving the DNA. For example, Epigenetic Remodeling factors, base editing enzymes, and transcriptional regulators can be delivered into the desired target site through its fusion into dCas9 (58). It was first shown in 2014 that the fusion of dCas9 to the Fok1 endonuclease creates a new gene editing tool with reduced off-target risk (59, 60). Fok1 has two subunits, each of which is fused to a separate dCas9. Each dCas9 binds to one of the two strands of the target sequence through distinct sgRNAs, leading to dimerization of Fok1 and cleavage of the target site (61, 62).

“Cas-CLOVER” is another gene editing system, based on fusing the naturally occurring Clostridium “Clo051” endonuclease with dCas9 (**Figure 2B**). The Dual-guided strategy for dimerization and activation of Clo051 increases the fidelity of Cas-CLOVER 25-fold greater than the classical CRISPR/Cas9 system (35). Cas-CLOVER generates DSB with large sticky ends which makes the site-specific insertion of genes more controllable (63). One of the distinct advantages of Cas-CLOVER over the classical gene editing tools is the possibility of gene editing in resting T cells which preserves the less differentiated phenotype of cells (35). P-BCMA-ALLO1 and P-MUC1C-ALLO1 are two allogeneic CAR T cell products developed by “Poseida Therapeutics” for the treatment of patients with multiple myeloma and MUC1C+ solid tumors, respectively. This company uses Cas-CLOVER to disrupt the T cell receptor B constant (TRBC) and B2M genes. These products are under evaluation in phase I clinical trials (NCT04960579, NCT05239143).

#### 2.2.3 LAGLIDADG homing endonucleases

LAGLIDADG homing endonucleases, commonly referred to as “Meganucleases,” represent a prominent group within the family of naturally occurring homing endonucleases, encompassing five distinct types. The term LAGLIDADG denotes a specific conserved amino acid sequence integral to the structural composition of these enzymes (64, 65). Unlike the modular design of Zinc Finger Nucleases (ZFNs), Transcription Activator-Like Effector Nucleases (TALENs), and the CRISPR/Cas9 system, meganucleases operate through a single protein that both recognizes specific DNA target sites and performs DNA cleavage, demonstrating a more streamlined mechanism of action. Although this non-modular architecture makes them difficult to re-engineer to target new sequences, they pose several merits that make them attractive tools for precise gene editing. For example, their small and non-modular structure, which is encoded by a single gene, facilitates their delivery by various delivery methods. Compared to ZFNs, TALENs, and CRISPR/Cas9, meganucleases recognize larger sequences (14–40 bp), resulting in high specificity cleavage with minimal off-target (25). While most of the meganucleases remain challenging to re-purposing, megaTAL, and ARCUS are two meganucleases-based tools that have been successfully used in CAR T cell therapy.

MegaTAL is an engineered nuclease made by fusing TALE arrays to the N-terminus of a meganuclease (**Figure 2C**). Meganucleases intrinsically have a low binding affinity to their target sequences. In megaTALs, the Low binding affinity of meganuclease to its target sequence is addressed by the binding affinity of TALE arrays. This unique structure sums up the site-specific binding of TALE domains and sequence-specific cleavage of meganucleases. MegaTAL activity requires the binding of both TALE and meganuclease domains to their target sites. This extended length of target sequence reduces the risk of mismatches and results in highly precise editing of target genes with minimal off-target effect (66–68).

Scientists of “Precision BioSciences” have developed a versatile genome editing tool called ARCUS. ARCUS is based on the naturally occurring I-Crel endonuclease. I-Crel is a homodimeric protein that is converted to a monomer in the ARCUS system (**Figure 2D**). Compared to other gene editing tools ARCUS has a very small size (1092 bp/40 kDa). Its small size and monomeric structure make it easy to vectorize and deliver to target cells. ARCUS provides the possibility of multiplex genome editing with close to zero off-target activity so that it can distinguish the target sequence from sequences that differ by only one base pair (69, 70). Anti CD19-PBCAR0191 (NCT03666000), anti-CD20 PBCAR20A (NCT04030195), anti-BCMA PBCAR269A (NCT04171843), and anti-CD19 PBCAR19B (NCT04649112) are four genome-edited CAR T cell products developed using ARCUS. In these products disruption of the TRAC gene has been coupled with site-specific insertion of the CAR into the TRAC locus. these products are being evaluated in phase I-II clinical trials.

#### 2.2.4 Base editors

The gene editing tools mentioned earlier function by creating DSBs at specified genomic locations. Research has indicated that DSBs can result in chromosomal abnormalities, including translocations (38, 71–73). In contrast, base editors represent an innovative category of gene editing tools capable of altering target genes without introducing DSBs (74). These tools are hybrids combining single-guide RNA (sgRNA) from the CRISPR system with a fusion protein. This protein fuses a deaminase enzyme to a Cas9 nickase (nCas9), enabling precise gene modifications without the risks associated with DSBs (**Figure 2E**) (75). By inducing a mutation in RuvC or HNH domains Cas9 only can nick one of the two DNA strands, while retaining its complementarity to sgRNA and PAM recognition properties (76). When the nCas9 nick the strand that is complementary to sgRNA (target strand), deaminase can modify basses in the other strand (non-target strand). The area that can be modified by deaminase activation is called the “base editing window” (77). There are two types of nucleotide deaminase, cytosine base editors (CBEs) and adenine base editors (ABEs) which remove the amine group from cytosine and adenine, respectively (74).

By removing an amine group, cytosine is converted to uracil. DNA polymerase reads uracil as thymine; therefore, cytosine is converted to thymine through DNA replication or repair. DNA repair machinery utilizes the base edited strand as a template to repair the nicked non-edited strand. So, the C: G base pair is converted to T: A (78). The cell-intrinsic “base excising repair pathway” can convert uracil back to thymine by uracil DNA N-glycosylase. In second-generation CBEs, attachment of a “uracil DNA N-glycosylase inhibitor” enzyme as a third moiety to the fusion protein leads to highly efficient base converting, three times more than last generation (79).

By the same mechanism, ABEs convert adenine to inosine, which leads to the conversion of the base pair A: T to G: C (80). In recent years, more advanced base editors have been introduced with the ability to convert cytosine to guanine (81) or simultaneous modification of cytosine and adenine (82). Base editors can be applied for creating point mutations, correction of single nucleotide variants, altering amino acid codons, introducing premature stop codons, and elimination of splice sites (12). BE-CAR7 is an allogeneic CAR T cell product developed using base editing strategy to disrupt *CD52*, *CD7*, and T cell receptor β constant (*TRBC*) genes. In a phase I clinical trial administration of BE-CAR7 showed promising results in three T-ALL patients (83).

Despite all the merits of base editors, some challenges limit their use. Although, base editors lack the DSBs-related side effects, however, off-target modifications can occur. Off-targets can be due to the mismatches between sgRNA and undesired sequence or due to the intrinsic affinity of deaminases for DNA (84). Base editing can only be used for single nucleotide variations. moreover, base editors are incapable of doing all 12 types of base-to-base conversions (79).

#### 2.2.5 Prime editors

Prime editors are newly emerged gene editing tools that make changes in target genes without generating DSB, similar to base editors. What sets prime editors distinct from base editors is the ability to perform all 12 possible base-to-base conversions and the possibility of small gene insertion (around 40 bp) or deletion (up to 80 bp in length). Prime editors can combine insertion, deletion, and base swapping (85). Additionally, in the context of gene insertion, prime editors are capable of inserting genes without the need for a donor DNA template, a distinct advantage over CRISPR-mediated gene insertion (86). Prime editing depends on two components: a prime editor (PE) that is made by fusing an nCas9 to a reverse transcriptase (RT), and a modified form of sgRNA called “prime editing guide RNA” (pegRNA). PegRNA is larger than traditional sgRNA (>100 nucleotides) and has a primer binding sequence (PBS) and a desired RNA sequence at the 3’ end (**Figure 2F**) (87).

When the pegRNA specifically recognizes and binds to its target DNA sequence, the nCas9 is activated to nick the DNA, creating a flap. Subsequently, the PBS on the pegRNA attaches to this DNA flap. The RNA sequence is then reverse-transcribed into DNA by RT. This newly synthesized DNA strand is fused with the nicked DNA strand, while the original, unedited segment of host DNA is removed by endonucleases (87). To correct the complementary, unedited strand of DNA, which does not match the newly edited strand, a second set of nCas9 and guide RNA is introduced, following the principles established by the third generation of prime editors. This approach ensures that nCas9 nicks the unedited strand, allowing the cell’s natural repair processes to use the edited strand as a template for repair. This results in two DNA strands that are now identical and correctly edited (88). Over time, prime editing technology has undergone three major iterations, with each version enhancing the system’s efficiency, binding accuracy, thermostability, and capability to correct mismatches. The most recent iteration incorporates these advancements, showcasing significant improvements in the technology’s performance and application potential (89, 90).

Although prime editors have shown encouraging results in primary cells and animal models, several limitations need to be addressed for their clinical translation. Most importantly the low efficiency of prime editing is the biggest limitation of this approach, which requires further modification and optimization of nCas9, RT, and pegRNA (91).

## 3 Gene editing strategies in CAR-T cell therapy

**Figure 3:**
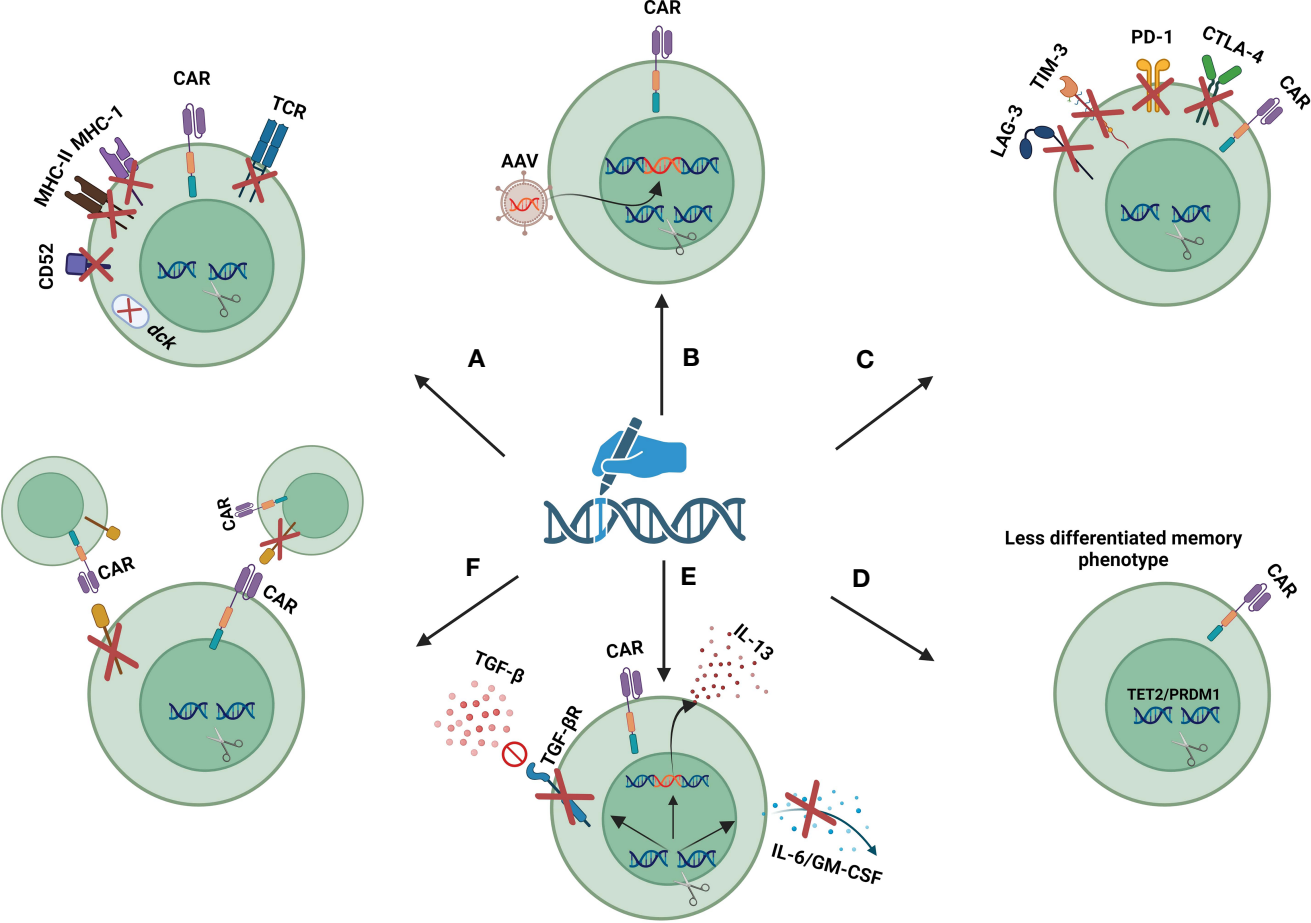
Gene editing strategies to improve safety and efficacy of CAR-T cells: **(A)** gene disruption targets for safe and efficient use of allogeneic CAR-T cells. **(B)** site-specific insertion of transgenes. **(C)** disruption of exhaustion markers. **(D)** disruption of epigenetic regulators to maintain the less-differentiated phenotype of CAR-T cells. **(E)** cytokine/cytokine receptor modulation. **(F)** disruption of fratricide-causing antigens. Created with biorender.com.

Irrespective of the type of used tool, gene editing technology has made it possible to further realize the therapeutic potential of CAR-T cells. In the following sections, different gene editing strategies to improve the safety and efficacy of CAR-T cell therapy are discussed (**Figure 3**).

### 3.1 Off-the-shelf CAR T cell production

The use of allogeneic sources of T cells to generate CAR T cells can overcome the limitations of autologous CAR T cell therapy, and provide a universal and integrated product that can be used for multiple patients (92). Allogeneic CAR T cell therapy offers many advantages, such as the use of high-quality healthy donor-derived T cells, the possibility of T cell phenotype selection, and the enabling of future re-administrations. Most importantly, by producing a readily available product the cost and time of treatment can be significantly reduced (93). Nevertheless, the risk of developing life-threatening GvHD and immunogenicity are the two main bottlenecks of allogeneic CAR-T cell therapy (94, 95). In recent years, gene editing strategies have emerged as a game changer in the field of allogeneic CAR-T cell therapy and have provided the ground for the safe use of allogeneic CAR-T cells.

The use of gene editing tools to disrupt the endogenous T cell receptor (TCR) has presented a strategy to circumvent GvHD in allogeneic CAR T cell therapy. This disruption involves selectively knocking out the genes responsible for the TCR α constant (TRAC) or TCR β constant (TRBC), which are essential for the production of the TCR α and β chains, respectively. Given that the TRBC gene has two potential constant regions, targeting the TRAC gene for disruption is generally preferred due to its simpler genetic structure (94). In 2012, Torikai and colleagues first demonstrated that the signaling from the endogenous TCR is not essential for the functionality of CAR-T cells. Their research showed that using ZFNs to disrupt the TRAC gene, responsible for TCR expression, does not compromise the anti-tumor efficacy of CAR-T cells (96). Subsequent research has supported these findings, further highlighting that TCR disruption significantly lowers the risk of GvHD (97, 98). several clinical trials are exploring the potential of TCR-disrupted allogeneic CAR-T cells (**Table 2**). Despite these advancements, there’s ongoing debate about the impact of TCR disruption. Some studies suggest that removing the endogenous TCR might adversely affect the persistence and proliferation of CAR-T cells in the body (99, 100).

Allogeneic CAR-T cells are rapidly rejected by host immune responses due to the MHC incompatibility between donor and recipient (101). This problem can be solved by removing MHC molecules from the surface of CAR-T cells. Since the genes encoding for MHC are highly polymorphic, direct disruption of individual MHC genes by gene editing tools is not possible. However, the elimination of MHC-I molecules can be achieved by knocking out the B2-microglobulin gene, a shared subunit between all MHC-I molecules (102). Moreover, MHC-II molecules can be removed through the disruption of CIITA and RFX genes, two master regulators of MHC-II genes (103, 104). Nevertheless, the risk of rejection is not completely eradicated by removing MHC molecules, as host natural killer (NK) cells may be activated against MHC-deficient cells (105). Therefore, in some allogeneic CAR-T cells, the removal of MHC molecules is coupled with the insertion of an NK inhibitory ligand. These inhibitory ligands include HLA-E-B2M fusion protein, E-cadherin, and siglec 7/9 (102, 106, 107). Studies have shown that the elimination of both MHC-I and MHC-II molecules in parallel with the insertion of an NK inhibitory ligand leads to more durability of allogeneic CAR-T cells (108). Another approach to increase the *in-vivo* persistence of allogeneic CAR-T cells is based on knocking out the *CD52* gene in CAR-T cells. In this manner by using an anti-CD52 monoclonal antibody host CD52^+^ lymphocytes are depleted, while CD52-negative CAR-T cells are not affected (109). Additionally, disruption of deoxycytidine kinase (*dck*), which is involved in fludarabine metabolism allows the use of fludarabine to deplete host lymphocytes (110).

### 3.2 Site-specific insertion of the gene of interest

Permanent CAR expression requires vectors capable of inserting the CAR transgene into the T cell genome. Lentiviral/retroviral (LV/RV) vectors are the most frequently used vectors in adoptive cell therapy, all approved CAR-T cells are produced using LV/RV vectors (1). LV/RV vectors randomly integrate into the host genome, which is associated with the risk of insertional mutagenesis. Moreover, there is the risk of other integration events such as reduced CAR expression (2) and vector-mediated clonal outgrowth (111, 112). In recent years Sleeping Beauty and piggyBac, two DNA transposon vectors, have been proposed as safe and efficient alternatives to viral vectors. These vectors can insert the desired transgene into the host genome by cut and paste mechanism. The safe integration profile of these vectors in preclinical studies opened the way to their clinical use (113). Early-phase clinical trials indicate that the utilization of transposon vectors yields levels of CAR expression that are comparable to those achieved by viral vectors, concurrently leading to substantial reductions in cost (8, 114). However, recent cases of T-cell lymphoma in some patients who received CD19 CAR T cells generated using PiggyBac transposon vector raise concerns about the mutagenicity of transposon vectors (115).

The findings underscore the need for advanced technologies that enable the precise and controlled integration of transgenes into designated safe locations within the host genome. This precision is achieved by leveraging gene editing tools to simultaneously disrupt a target gene and insert a new sequence at the site of disruption (27). The process involves creating a double-strand break (DSB) at a specific genomic site with site-specific nucleases. In the presence of a donor DNA template, which includes sequences homologous to the regions flanking the DSB, the cell’s HDR mechanism uses this template to mend the break, thereby integrating the new sequence at the precise location. Adeno-associated viral (AAV) vectors, known for their safety in human applications, can be utilized to deliver the donor template into the cells (116). Examples of this technology in action include the insertion of a CAR construct into the TRAC/TRBC locus, the integration of the B2M-HLA-E fusion peptide gene at the β2-microglobulin locus, and the incorporation of the interleukin-15 coding sequence at the interleukin-13 gene locus to improve persistence and effectiveness of CAR-T cells. These strategic insertions, demonstrated in various studies, have been successfully employed to enhance the specificity and efficacy of gene editing applications in research and therapeutic contexts (117, 118).

In addition to coupling the disruption of one gene with the insertion of another gene, this strategy offers many other advantages. Site-specific insertion of CAR eliminates the risk of insertional mutagenesis. Moreover, the insertion of a transgene into the appropriate site leads to an integrated expression pattern. For example, the insertion of a CAR coding sequence at the TRAC/TRBC locus puts the CAR under the control of endogenous TCR regulatory elements and leads to delayed exhaustion of CAR-T cells by preventing tonic signaling (2). In addition to the TRAC/TRBC locus, there are several potential safe harbors for site-specific insertion of CAR such as TET2, PCD1, CCR5, AAVS1, and ROSA26 locus (2). Additionally, since the gene editing efficiency is not 100%, the site-specific inserted gene (if it has surface expression) can be used as a selective marker for quantitation of gene editing efficiency and further purification of genome-edited CAR-T cells.

### 3.3 Prevention of exhaustion

The advancement of CAR-T cell therapy for solid tumors is hindered by multiple obstacles, including ineffective trafficking and penetration of CAR-T cells into tumor sites, their diminished longevity, and the suppressive nature of the tumor microenvironment (119). Notably, the interaction between the PD-1 receptor on CAR-T cells and the PD-L1 ligand on tumor cells is known to contribute to CAR-T cell exhaustion. Furthermore, research has demonstrated that combining CAR-T cell therapy with monoclonal antibodies targeting PD-1 or PD-L1 can significantly enhance the persistence, anti-tumor efficacy, and delay the exhaustion of CAR-T cells *in vivo* (120). However systemic administration of anti-PD-1/PD-L1 monoclonal antibodies comes with some adverse effects, such as the development of antidrug resistance, capture of antiPD-1 monoclonal antibodies before reaching the T cell, and enhanced activation of autoreactive T cells (119). Several *in vitro* and *in vivo* preclinical studies have demonstrated the superior safety of PD1-disrupted CAR-T cells compared to the combination of CAR-T cell therapy and anti-PD1/PD-L1 monoclonal antibodies. These studies also show that disruption of PD-1 leads to increase *in vivo* persistence and antitumor activity of PD1-disrupted CAR-T cells compared to the same non-manipulated CAR-T cells (119, 121, 122). Based on these findings several PD1-disrupted CAR-T cells are being evaluated in clinical trials (**Table 2**). Although disrupting PD-1 enhances the durability of CAR-T cells, it does not entirely eliminate their exhaustion (123). Intriguingly, research by Odorizzi PM and colleagues has revealed that PD-1 plays a crucial role in preventing the overstimulation, excessive proliferation, and terminal differentiation of T cells. Therefore, disrupting PD-1 might inadvertently impair the long-term effectiveness of CAR-T cells used in adoptive cell transfer therapies (123). This underscores the need for further research to thoroughly understand the implications of PD-1 gene disruption for therapeutic applications.

Expression of CTLA-4, LAG3, and TIM3 are other hallmarks of T cell exhaustion. In contrast to PD-1 which inhibits T cell activity in the late phases of immune response, CTLA-4 inhibits T cell function in the earlier phases. In a preclinical study, it was shown that CRISPR/Cas9 mediated disruption of CTLA-4, but not PD-1, restored the function of patient-derived CAR-T cells that are poorly fit due to the immunosuppressive effects of cancer cells and anticancer therapies (124). Also, disruption of LAG-3 using CRISPR/Cas9 has been successfully performed without affecting the viability and phenotype of CAR-T cells during culture (124). Ciraolo et al. (125) revealed that simultaneous disruption of PD-1, LAG-3, and TIM-3 in CD8+ T lymphocytes promotes the persistence and proliferation of adoptively transferred T cells.

The underlying mechanisms of exhaustion in T cells are very complex and many other factors can negatively affect the persistence of CAR-T cells. Gene editing has been successfully used in various studies to disrupt negative regulators of T cells. These negative regulators include Cytokine-inducible SH2-containing protein (CISH) (126), DNA methyltransferase 3 alpha (DNMT3A) (127), Cbl-b (128), nuclear receptor transcription factors NR4A (129), Diacylglycerol kinases (130), adenosine A2A receptor (131) ID3, SOX4 (132), TGF-β receptor II (133), and protein tyrosine phosphatase 1B (PTP1B) (134).

### 3.4 Fratricide prevention

CAR-T cell therapy against antigens that have shared expression between CAR-T cells and tumor cells is limited by the risk of fratricide or self-killing of CAR-T cells during culture or *in vivo* activity. This is more highlighted in T-cell malignancies, where all targetable antigens are shared between malignant cells and CAR-T cells (135). CD7, CD2, TCRαβ/CD3, and CD5 are favorable CAR-targeted antigens for elimination of malignant T cells, but their shared expression between effector and target cells is a key limiting factor in CAR T cell therapy of T cell malignancies (83). Gene editing tools provide the ability to knock out the fratricide-causing antigens to prevent self-killing of CAR-T cells. Furthermore, by simultaneously disrupting the TCR and fratricide-causing antigens a bank of universal CAR T cells with different specificities can be created. So, treatment with different CAR-T cells with different specificities can be combined to complete the elimination of malignant cells and the prevention of antigen loss (83). WU-CART-007 (NCT05377827, NCT04984356) and CD7 UCAR-T cell (NCT04264078) are two universal CD7 CAR T cells being evaluated in phase I clinical trials. These two products utilize CRISPR/Cas9 to disrupt *TRAC* and *CD7* genes. CT125A is a universal CD5 CAR T cell that uses CRISPR/Cas9 to disrupt *CD5* and *TRAC* genes. These products are under investigation in a phase I clinical trial for patients with CD5+ hematologic malignancies (NCT04767308). BE-CAR7 is an anti-CD7 allogeneic CAR-T product that utilizes base editing to disruption of *CD7*, *TRBC*, and *CD52* genes. In a recent phase I clinical trial (ISRCTN15323014) treatment with BE-CAR7 showed promising results in 3 patients with T-cell acute lymphoblastic leukemia (136).

Fratricide of CAR-T cells is not exclusive to T-cell malignancies and can happen in other cases as well. CS1 is an antigen expressed by myeloma cells in different subgroups of multiple myeloma (137), so it is a potential target for CAR design. CS1 is not expressed by hematopoietic stem cells and most of the normal cells, but NK cells and T cells express CS1 at a low level (138). UCARTCS1 is an allogenic CAR-T cell developed by “Cellectis” for patients with relapsed/refractory multiple myeloma (NCT04142619). The company has used TALENs-mediated disruption of the CS1 gene to prevent fratricide in UCARTCS1 product (139). UCARTCS1 has shown favorable antileukemic activity in preclinical studies (140).

CD38 is a surface marker mainly expressed by plasma cells (141). It is also revealed on the surface of natural killer cells, T cells, and B cells (141). Overexpression of CD38 is seen in various hematological malignancies, especially in multiple myeloma (142). Therefore, CD38 is a potential marker for targeted therapy of hematologic malignancies. For example, “Daratumumab” is an FDA-approved anti-CD38 monoclonal antibody for the treatment of multiple myeloma (143). Since CD38 is expressed by NK cells and T cells, engineering NK cells and T cells with CD38 CAR can lead to self-killing of CAR NK and CAR T cells (141, 144, 145). Therefore, gene editing tools can be used to prevent self-killing of CD38 CAR T cells.

Fusing the natural killer group 2D (NKG2D) receptor with the CD3ζ domain of the TCR creates a CAR capable of recognizing multiple stress-induced ligands present on a wide array of hematologic malignancies and solid tumors (146). However, the activation of CAR-T cells induces a stress response that leads to the transient expression of NKG2D ligands on the CAR-T cells themselves, raising the risk of fratricide. This issue poses a challenge to the large-scale production of NKG2D CAR T cells (147). Celyad Oncology has developed CYAD-02, an NKG2D CAR T cell product, employing shRNA) to suppress the expression of MIC-A and MIC-B, which are primary NKG2D ligands, to mitigate the risk of fratricide (148). While shRNA can effectively downregulate these ligands, gene editing technologies offer a more efficient solution for inhibiting gene expression. Despite their potential, gene editing tools have yet to be applied to prevent fratricide in NKG2D CAR T cell development.

### 3.5 Miscellaneous strategies

The scope of using gene editing technologies in CAR-T cell therapy is expanding and its applications are increasing in preclinical and clinical studies. Gene editing technologies can be used to render CAR-T cells resistant to inhibitory cytokines or make them capable of producing a specific beneficial cytokine. For example, CRISPR/Cas9-mediated disruption of TGF-β receptor II has led to increased durability and anti-tumor response of CAR-T cells (133, 149). Genetic ablation of GM-CSF and IL-6, two important mediators of cytokine release syndrome and neurotoxicity, can promote CAR-T cell function and inhibit the occurrence of cytokine release syndrome and neurotoxicity (150, 151). CRISPR/Ca9 has been successfully used for site-specific insertion of IL-15 into the IL-13 gene locus. Given that IL-13 is highly expressed in activated T cells, this strategy puts the IL-15 gene under the control of hyperactive IL-13 promoters, leading to more IL-15 production by CAR-T cells (118).

Disruption of epigenetic regulators by gene editing tools can lead to epigenetic remodeling and affect the function and persistence of CAR-T cells. Disruption of TET2 and PR domain finger protein (PRDM1) has been shown to result in a sustained antitumor response of CAR-T cells by maintaining their less differentiated phenotype (152).

### 3.6 IPSC-derived CAR-T cells

The introduction of induced pluripotent stem cells (IPSCs) has opened a new way in the field of cellular therapies since IPSCs can be generated by reprogramming any somatic cells. In 2006 Dr. Yamaka showed that by introducing four transcription factors, including Oct3/4, Sox2, c-Myc, and Klf4, somatic cells are reprogrammed into stem cells, whose potency is on par with embryonic stem cells (ESCs), and can be differentiated into desired lineage (153).

The use of IPSCs in CAR-based immunotherapy is associated with several advantages over the conventional primary cell sources. Feasibility of gene editing in IPSCs and their differentiation into T cells leads to the formation of a uniform final product which helps standardization of drug dosing and treatment approach (26). In addition to CAR αβ-T cells, IPSCs can be differentiated into any hematopoietic cell type such as CAR-NK, CAR γδ-T, CAR-macrophages, and CAR-neutrophils (154–156). The use of IPSCs to generate CAR-T cells circumvents the requirement for long-term ex vivo expansion and cultivation of CAR-T cells, which results in the prevention of long-term cultivation-associated limitations such as terminal differentiation of CAR-T cells (157).

Currently, several biotechnology companies are working on developing off-the-shelf iPSC-derived cell therapy products and some of these products are being evaluated in phase I clinical trials. As one of the leading companies in the field of CAR-IPSCs “Fate therapeutics” has developed two IPSC-derived CAR-T (iCAR-T) and three IPSC-derived CAR-NK (iCAR-NK) products which are under assessment in phase I trials. The company’s CAR-IPSC-derived products pipeline comprises FT-819 (CD19 iCAR-T) (NCT04629729), FT-825 (HER2 iCAR-T) (NCT06241456), FT-522 (CD19/CD20 dual targeting iCAR-NK) (NCT05950334), FT-576 (BCMA iCAR-NK) (NCT05182073), FT-596 (CD19 iCAR-NK) (NCT04245722). FT-819 are generated by CRISPR/Cas9-mediated insertion of a novel 1XX CAR transgene into the TRAC locus. In stringent xenograft models, FT-819 showed higher anti-tumor potency than conventional primary CAR-T cells. recently the interim results of FT-819 have been published which indicate its safety with no cases of ICANS, GvHD, dose-limiting toxicity, or Grade ≥III CRS (158). The interim results of FT-576 have also been published recently which indicate the safety of FT-576 in all treated patients with no cases of CRS, GvHD, ICANS, or dose-limiting toxicity (159).

“Century Therapeutics” is another company that is assembling a robust portfolio of iPSC-derived NK, γδ and αβ T cell therapy. Among the products of this company “Cnty-101” -an iPSC-derived CD19 CAR-NK- has been entered to clinical phase and is currently being evaluated in phase I ELiPSE-1 trial (NCT05336409). Cnty-101 harbors six genetic engineering including transduction to express CD19 CAR, IL-15 transgene, EGFR safety switch, and gene editing to eliminate both MHC-I and MHC-II and insertion of a HLA-E-β2M. Recently results of treating a 63-year-old case of follicular lymphoma with Cnty-101 have been released by Century Therapeutics which indicate safety and anti-tumor activity of Cnty-101 (160).

Nonetheless, several bottlenecks should be considered for the clinical translation of IPSC-derived products. Most importantly it should be noted that the use of IPSC-derive cells is associated with the risk of tumorigenicity and immunogenicity, underscoring the need for precise quality control and safety assessment before their release (161). Moreover, IPSC-differentiation toward T cells by current methods leads to an imbalance between CD4+ and CD8+ T cells, and CD4+ cells are rarely seen within IPSC-derived T cells. Since the imbalance between helper T, and cytotoxic T cells can adversely affect the therapeutic efficacy of CAR-T cells, optimization of IPSC differentiation methods is needed (162).

## 4 Considerations and risk assessment in genome-edited CAR-T cell therapy

### 4.1 Genotoxicity

The application of gene editing technologies, while transformative, is not without risks, notably the potential for off-target and on-target genotoxicity. A significant incident occurred on October 7, 2021, when the FDA paused all clinical trials involving genome-edited allogeneic CAR-T cells by Allogene Therapeutics following a reported chromosomal abnormality in a patient treated with the ALLO-501A product (163). By January 2022, the FDA lifted this hold after investigations concluded that the chromosomal irregularity was not linked to the TALEN-mediated gene editing technique (164, 165). To date, there have been no reported clinical adverse effects directly tied to gene editing. However, the presence of chromosomal abnormalities in edited cells underscores the necessity for ongoing monitoring of patients who undergo these therapies. Recently, several cases of secondary malignancies have been reported as a result of autologous CAR-T cell therapy, raising concerns about the safety of CAR-T cell therapy (166). Even more concerns would be expected from utilizing genome-edited allogeneic CAR-T cells. This emphasizes the importance of long-term follow-up of treated patients.

According to the recently released FDA industry guidance document on human gene therapy products incorporating human gene editing, the specific risk associated with genome-edited cell products are the risk of off-target activity of nucleases, unintended consequences of on-target editing including chromosomal translocations, and the unknown consequences of on- and off-target editing. The FDA recommends that release testing of *ex vivo* genome-edited cell products should include evaluation of on-target editing efficiency, assessment of editing events at the target site, assessment of the frequency of off-target events, determining the total number of edited cells, assessing intrachromosomal and interchromosomal rearrangements, and measuring residual components of gene editing tools. Lot release testing should also have strict acceptance criteria for the quantity of potentially alloreactive T cells and the lack of aberrant growth. For clinical trials, a precise safety monitoring strategy should be provided with a toxicity grading system and toxicity management plan. Patients should be followed up for adverse consequences of on-target editing, aberrant cellular and chromosomal changes, immunogenicity, and tumorigenicity. As the long-term consequences of on-target editing are now unknown, the FDA recommended long-term follow-up of treated patients for up to 15 years (167).

Genome editing can yields several unintended genomic alterations including small insertion/deletion (in/del) mutations, large deletions, chromosome rearrangement, loss-of-heterozygosity (LOH), chromothripsis, or even loss of whole chromosome (168). Even in the case of on-target cleavage genotoxicity is possible when the created DSB interacts with other nuclease-induced DSB (in multiplex strategy) or a spontaneous DSB within the genome. Thus, performing preclinical safety assays is necessary for developing genome-edited CAR-T products (40). The presence of 0.1% cells with unintended genetic alterations in the infused product can translate to 10^5^ – 10^7^ mutated cells after *in vivo* expansion in recipients (168). Nonetheless, there is no single unbiased genome-wide method to detect the whole spectrum of genetic alterations. Due to the limited capacity of various analyzing techniques, it is important to combine various techniques to ensure the maximum detection of structural variants and in/del mutations (169).

For detecting unintended genetic modifications, several methods exist, ranging from in silico, in cellula, to *in vitro* approaches. In silico prediction tools offer a cheap and fast method for the prediction of off-target sites. These methods are based on the similarity of the gRNA spacer and the human genome; however, this approach yields a high rate of false-positive results (170). The most frequently used method to analyze gene editing is the generation of short amplicons with a length of less than 1kb using polymerase chain reaction (PCR) and their sequencing by Sanger sequencing or next-generation sequencing (NGS). Nonetheless, using this approach, only mutations located in small amplicons can be detected and many structural variants may not be detected (169). Amplicons of regions of interest can also be analyzed by a non-sequencing technique called T7 Endonuclease 1 (T7E1) Assay. Although the T7E1 technique can detect the presence of nuclease-induced mutations, however, it is non-quantitative and does not reveal the sequence difference of various in/del mutations (171). Among all the assays, NGS is the only method that offers high-resolution qualitative and quantitative data Across the whole spectrum of modifications. NGS is applicable in various stages of the gene editing process including detection of off-target by whole genome sequencing or confirming on-target edits by targeted sequencing (169). whole genome sequencing (WGS), Whole exome sequencing (WES), and total RNA sequencing (RNA-seq) can be used for the detection of in/del mutations and structural variants; however, it should be considered that compared to WGS, WES, and RNA-seq have a limited scope since they only analyze protein coding region (~1−2% of the human genome). On the other hand, WGS lacks the adequate sensitivity to detect low-frequency genetic alteration in a pooled cell population (169). Techniques such as GUIDE-seq, E-CRISP, Digenome-seq, SITE-seq, CIRCLE-seq, DISCOVER-seq, CHANGE-seq, End-seq, Digenome-seq, IDLV, capture, ITR-seq, BLESS, BLISS, and ChIP-seq are among the most utilized for sequencing analysis (170). Large deletions can be detected by third-generation sequencing techniques including Oxford Nanopore and PacBio. These techniques directly sequence a single DNA molecule without the need for breakdown or amplifying the DNA (168). single-cell RNA sequencing (scRNA-seq) can provide more precise quality control of edited products, but its application in CAR-T cell therapy, where millions to billions of edited cells are infused into patients, is challenging (168).

The risk of translocations becomes particularly pronounced with the use of multiplex gene editing strategies, highlighting the critical need for precision and caution in these interventions. It has been reported that Multiplex gene editing in T-cells yields translocations at a ∼1% frequency. Although the number of edited cells with chromosomal rearrangements reduces after CAR-T infusion and to date no clinical consequences have been reported, analyzing the generated rearrangement is necessary (38). The major drawback of the use of conventional PCR-based methods including quantitative PCR (qPCR) and droplet digital PCR (ddPCR) for detecting translocations is that they are primer-based and need preexisting knowledge about exact translocations and in the case of extensive end processing they may underestimate translocation frequency. Directional Targeted Sequencing (UDiTaS) or high throughput genome-wide translocation capture offer unbiased translocation detection, however, they are also primer-based and are subject to primer loss. Among the non-PCR-based methods, fluorescence *in situ* hybridization (FISH) allows the detection of both known translocations (interphase FISH) and unbiased translocation (metaphase-FISH), however low throughput and expensive procedure are the main bottlenecks of FISH (172). Megadeletions and loss of heterozygosity (a genetic abnormality in which diploid cells lose one of the two copies of a genomic segment) can be detected by single-nucleotide polymorphism (SNP) genotyping-based tools, digital karyotyping, or quantitative genotyping PCR (qgPCR) (173, 174). To ensure full capture of chromosomal abnormalities it is rationale to combine several methods.

Considering the impossibility of evaluating all edited cells and the technical limit of molecular methods, it is necessary to combine molecular assays with functional assessments (175). Currently, *in vivo* tumorigenicity assays are widely used to evaluate tumorigenicity of genome edited cells. In this method, genome-edited cells are injected into mice with severe combined immunodeficiency, and the treated mice are followed up for 6–12 months. According to the definition of the World Health Organization (WHO), tumorigenicity is “the capacity of a cell population inoculated into an animal model to produce a tumor by proliferation at the site of inoculation and/or at a distant site by metastasis” (176). Nonetheless, this approach suffers from variable engraftment rates (ranging from 25%-80%) of different cell populations in immunocompromised mice and is a time-consuming process (177). Recently, *in vitro* transformation assays (such as soft agar colony-forming assay and growth in low attachment assay) have been developed to remove the challenges of *in vivo* tumorigenicity assays. *In vitro* transformation, assays are based on the monitoring of malignant transformation-induced phenotypic changes such as the acquisition of anchorage-independent growth or disorganized pattern of colony growth. Accumulated evidence indicates that the underlying mechanisms of *in vitro* transformation of cells are similar to those of *in vivo* tumorigenesis (177, 178).

### 4.2 Type of used vector

The use of viral vectors or plasmid vectors leads to the permanent or long-term expression of nucleases which increases the risk of genotoxicity and DNA damage response (40, 179). Additionally, it requires nuclease transcription and translation which delays the editing process (11). The use of mRNA is another option, which yields transient expression of nucleases and reduces the editing time by eliminating the need for transcription. Nevertheless, the lower stability of mRNA is the main bottleneck of this method (11). CRISPR/Cas9 and other CRISPR/based systems can be delivered into the cells in the form of ribonucleoprotein (RNP), which can unlock the bottlenecks of Cas9 delivery by other vectors. The RNP complex can perform gene editing immediately after entering the cells which substantially facilitates the editing process (11). Nevertheless, some Cas nucleases perform poorly when utilized in RNP format (180).

### 4.3 Editing efficiency and purification of edited cells

Finally, it should be noted that the efficiency of none of the above-mentioned gene editing tools is 100% and a significant percentage of unedited cells remain in the final product. For instance, in the first use of ZFN to remove TCR that was conducted in 2012, electroporation of ZFN mRNA to target *TRAC* or *TRBC* led to disruption of TCR in 60% and 20% of cells, respectively (96). In UCART19 trial TALEN-mediated disruption of *TRAC* and *CD52* genes led to depletion of both TCRαβ and CD52 from more than 64% of cells (72). The efficiency of CRISPR/Cas9 in the disruption of *TRAC* and insertion of CAR construct within the disrupted locus was also reported as 70% (97). While advanced gene editing tools significantly reduce off-target events, the editing efficiency is not considerably different from classical tools. The efficiency of TCR disruption using megaTAL and ARCUS was reported as around 40% and 60%, respectively (67, 116). This value for Cas-clover and base editors has been reported in clinical trials as a variable percentage between 50% to near 100% and 62% to near 100%, respectively (35, 136). The released results indicate that irrespective of used gene editing tools, up to 50% of T cells may remain unedited which is a considerable amount. This means that around half of infused allogeneic CAR-T cells may have allogeneic properties which can lead to GvHD development or attenuating therapeutic efficacy due to the rejection of MHC-positive CAR-T cells. According to the experience of haploidentical stem cell transplantation, to prevent GvHD, the number of αβ-TCR+ T cells should not exceed 5x10^4^ per kilogram of body weight (181, 182). This underscores the need for an additional step to remove unedited cells to obtain a unified and pure product. In 2020, Juillerat et al, have developed a novel Straightforward approach to produce ultrapure TCRαβ-negative CAR-T product. In this method, 48h after genome editing in T cells, an anti-CD3 CAR construct is transiently expressed in CAR-T cells through electroporation of CD3 CAR mRNA, which leads to the killing of the residual TCRαβ+ CAR-T cells. Using this approach the final purity of 99–99.9% was achieved without affecting production yield or cell fitness (183). Nonetheless, the most frequently used approach to deplete unedited cells in clinical trials is based on the use of commercially available and cost-beneficial magnetic bead-mediated depletion systems. CliniMACS devices (Miltenyi Biotec, Bergisch Gladbach, Germany) can reduce TCRαβ+ to about 0.00097% without significantly affecting cell viability (184). In the UCART19 trial, CliniMACS-mediated depletion of TCRαβ+ T cells led to a final product with less than 0.7% TCRαβ+ CAR-T cells (72). In case of HLA-I disruption, to prevent allorejection of allogeneic CAR-T cells the number of HLA+ cells must be decreased to 1 in 10^3^-10^4^ cells (102). It has been reported that after ZFN-mediated disruption of *β-2M*, the use of CliniMACS devices could increase the purity of HLA-I-negative CAR-T cells from less than 52% to more than 95% which can efficiently prevent CD8+ T cell-mediated rejection of allogeneic CAR-T cells (102). Thus, MACS (Magnetic-Activated Cell Sorting) systems provide an efficient and cost-beneficial approach to purifying genome-edited CAR-T cells.

## 5 Conclusions

While CAR-T cell therapy has revolutionized cancer treatment, it encounters several unresolved obstacles. Recent advancements in gene editing have offered promising solutions to these challenges. Gene editing techniques have been effectively employed to eliminate specific genes that undermine the persistence and functionality of CAR-T cells. Notably, gene editing is facilitating the development of “off-the-shelf” CAR-T products, making these therapies more readily available. Additionally, gene editing enables the precise insertion of target genes directly into specific genomic locations, marking a significant stride towards establishing a viral-free method for gene integration.

The clinical use of gene editing tools is increasingly expanding and the number of clinical trials with genome-edited CAR-T cells indicates a bright future for these products. Nevertheless, there are few available clinical data about the efficacy and safety of genome-edited CAR-T cells. Moreover, the genotoxicity of programmable nuclease and the long-term consequences of disrupted genes remain a concern. This highlights the need for more clinical trials with genome-edited CAR-T cells and the necessity for long-term follow-up of treated patients. It is hoped that further modifications and optimizations of gene editing tools and their delivery methods will facilitate the safe and efficient clinical use of gene editing technology in CAR-T cell therapy.

## Author contributions

VM: Investigation, Methodology, Writing – original draft. EK: Investigation, Methodology, Writing – original draft. MA: Investigation, Writing – original draft. AO: Supervision, Validation, Writing – review & editing. NA: Conceptualization, Supervision, Writing – review & editing.
